# Advancements in the Use of Hydrogels for Regenerative Medicine: Properties and Biomedical Applications

**DOI:** 10.1155/2022/3606765

**Published:** 2022-11-07

**Authors:** Andrea Revete, Andrea Aparicio, Bruno A. Cisterna, Javier Revete, Luis Luis, Ernesto Ibarra, Edwin A. Segura González, Jay Molino, Diego Reginensi

**Affiliations:** ^1^Biological Engineering, Faculty of Biosciences and Public Health, Universidad Especializada de las Americas (UDELAS), Panama City, Panama; ^2^Biomedical Engineering, Faculty of Health Sciences and Engineering, Universidad Latina de Panama (ULATINA), Panama City, Panama; ^3^Department of Neuroscience and Regenerative Medicine, Medical College of Georgia, Augusta University, Augusta, GA, USA; ^4^Experimentia S.A, Development of Innovative Strategies in Biomedicine and Sustainable Development, Panama, Panama; ^5^Instituto Técnico Superior Especializado (ITSE), Panama, Panama; ^6^Integrative Neurobiology, School of Medicine, Universidad de Panama (UP), Panama, Panama; ^7^Center for Biodiversity and Drug Discovery, INDICASAT-AIP, City of Knowledge, Panama, Panama

## Abstract

Due to their particular water absorption capacity, hydrogels are the most widely used scaffolds in biomedical studies to regenerate damaged tissue. Hydrogels can be used in tissue engineering to design scaffolds for three-dimensional cell culture, providing a novel alternative to the traditional two-dimensional cell culture as hydrogels have a three-dimensional biomimetic structure. This material property is crucial in regenerative medicine, especially for the nervous system, since it is a highly complex and delicate structure. Hydrogels can move quickly within the human body without physically disturbing the environment and possess essential biocompatible properties, as well as the ability to form a mimetic scaffold *in situ*. Therefore, hydrogels are perfect candidates for biomedical applications. Hydrogels represent a potential alternative to regenerating tissue lost after removing a brain tumor and/or brain injuries. This reason presents them as an exciting alternative to highly complex human physiological problems, such as injuries to the central nervous system and neurodegenerative disease.

## 1. Introduction

Biomaterials are materials designed to interact with biological systems to evaluate, treat, or substitute any tissue, organ, or function in a living organism. These can interact with a biological system in various applications in biomedicine [1, 2]. The purpose of biomaterials is to interact with the living organism; that is, contact with the biological system. Thus, some biomechanical requirements must be met: (i) biocompatibility, which is the ability of the material to induce the least immunological rejection possible with the human body functioning harmoniously in synchronization with the host; (ii) adequate durability related to function; (iii) capacity to be bioreabsorbed (the body can metabolize it); (iv) biodegradability, which is the capacity of biological degradation; (v) desired mechanical properties depending on stresses and deformations that may occur in it; and (vi) they must not show toxic effects, the material must not be toxic or carcinogenic since it will be in contact with living organisms [3].

Biomaterials can be classified into synthetic and natural [4]. Among synthetics, we can find metals, ceramics, polymers, and composite materials [5]. These materials can be produced under controlled conditions; therefore, their mechanical and physical properties can be predicted. However, a disadvantage of synthetic biomaterials is their reduced biocompatibility and their low ability to induce tissue regeneration [6]. On the other hand, natural biomaterials present better scaffold-cell interactions due to their specific molecular domains but are more complex in their molecular architecture [7]. Natural biomaterials can be protein-based and generally obtained from animal and human sources. They include bioactive molecules that mimic the extracellular environment (e.g., collagen, fibrin, gelatin, and keratin) or polysaccharide-based, mainly obtained from algae (alginate, agarose, galactans, and carrageenan), higher plants (cellulose, pectin, guar gum, and starch), microorganisms (xanthan gum, dextran, gellan gum, pullulan, and bacterial cellulose), and animals (hyaluronan, chondroitin, heparin, chitin, and chitosan) [8–11].

Natural biomaterials present a crucial subset of biomaterials for use as tissue engineering scaffolds due to their high bioactivity, biocompatible character, mechanical kinetics, and degradation properties, and their intrinsic structural resemblance to the extracellular matrix (ECM) of native tissue; furthermore, in their application in biological systems, they do not release cytotoxic by-products during their degradation. In addition, one of the main advantages of natural biomaterials is their innate ability to promote biological recognition, as they can positively support various cellular processes [6]. There is evidence that natural biomaterials, based on decellularized extracellular matrix (dMEC), which preserve native tissue properties and promote functional regeneration of injured tissue, can be used in various preclinical applications [12–14]. The tissue decellularization process mainly eliminates a specific tissue's cellular content, preserving the extracellular matrix's structure and specific chemical signals of the extracellular matrix [15]. dMEC has been shown to provide tissue-specific properties that influence multiple cellular processes, such as chemotaxis, mitogenesis, and differentiation [16]. In addition, recent advances in this field have shown that the development of injectable decellularized biomaterials can serve as protective vehicles for cells and trophic factors and promote *in situ* regeneration of damaged tissue [17]. These new applications of decellularized biomaterials implanted at the site of trauma or disease can promote the generation of a reparative niche that could lead to local tissue repair [18].

The biocompatibility and biofunctionality of implantable biomaterials remain significant challenges for their wide use in biological applications. Furthermore, they should come into direct contact with tissues and fluids. Therefore, the processes of immunological and biological response must be considered (e.g., inflammation in the receiving organism) [19]. Cell-biomaterial interaction is critical in integrating implants based on biomaterials, as it precedes cell proliferation, cell migration, and cell differentiation [20]. Cell adhesion is mediated by serum proteins adsorbed on the surface of the material, such as immunoglobulins, vitronectin, fibrinogen, and fibronectin, in addition to the biomechanical characteristics of the material [21]. The interaction between the cells and the biomaterial implies a series of molecular events that occur both at the intracellular level that promote the expression of various transcription factors and gene regulation, as well as at the extracellular level mediated by multiple signals present in the ECM [22].

Cell adhesion is essential in cell viability studies since cell dispersion, migration, and differentiation depend on it. Therefore, controlling the interaction between cell receptors and the biomaterial surface is probably one of the most critical factors for designing biocompatible biomaterials in biomedical applications [23]. The process of cell adhesion to the surface of a biomaterial is thanks to adhesion proteins (fibronectin, laminin, and collagen) that use specific cell receptors found in the cell membrane (integrins). Integrins are responsible for recognizing adhesion proteins and facilitating their communication to achieve the adhesion process [24]. Biomaterials conditioned to meet specific topographical characteristics (porosity, stiffness, and roughness) allow cells to be guided or instructed in their expansion and development. Through cell studies, it has been possible to know that roughness is significant in the cell adhesion process, and it has been shown that this process, together with cell proliferation, is directly proportional to the level of roughness of the biomaterial surface [25].

**Figure 1:**
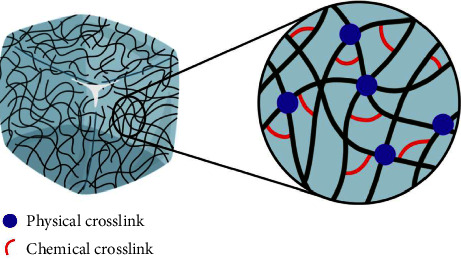
Hydrogel structure.

Among the most widely used biomaterials in biomedical studies for tissue regeneration are hydrogels, which correspond to polymeric networks with high water absorption capacity; cross-linking changes a liquid polymer to a solid or gel by modifying the movement of the molecules that form the hydrogel. When polymer chains cross-link, they lose some of their ability to move as individual polymer chains. Their biomedical applications are diverse, highlighting their role as scaffolds in tissue injuries and sustained drug release systems. They are also three-dimensional scaffolds for various cell therapies, providing a microenvironment with biomimetic properties that promote the recovery of damaged tissue [26, 27] (Figure 1).

## 2. Basic Concepts of Hydrogels in Biomedicine

Hydrogels have been highly studied and used due to their many characteristics. First, we can refer to their capacity for high water content. Water must constitute at least 10 to 20% of the total weight (or volume) for a material to be a hydrogel; this property allows hydrogels a degree of flexibility similar to natural tissue in various applications in biomedicine [28]. This capacity is due to its hydrophilic structure, which allows water to be conserved within its three-dimensional networks [29]. Indeed, hydrogels have the advantages of higher biocompatibility, biodegradability, adequate mechanical strength, a porous structure, and excellent ability for both adsorption and diffusion of water [30]. However, its potential in various biomedical applications is sometimes hampered by its low mechanical resistance and high electrical and thermal capacity [31]. Over the years, it has been possible to use scaffolds made from hydrogels to treat various types of tissues: cartilage, bone, muscle, fat, liver, and neurons [32].

### 2.1. Classification of Hydrogels

**Figure 2:**
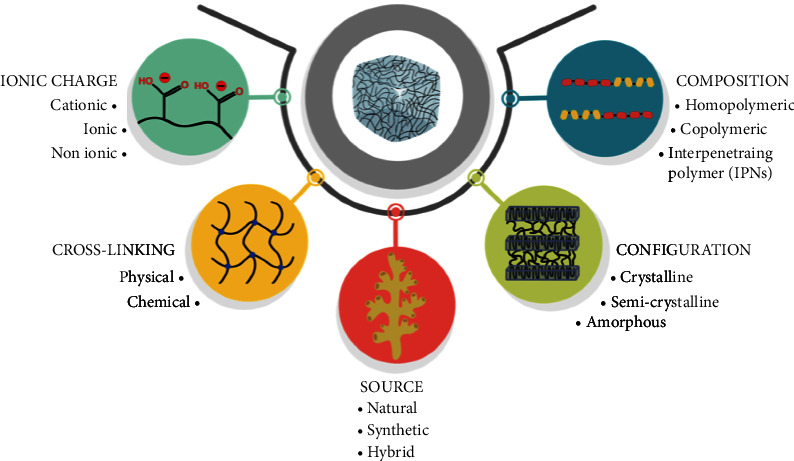
Hydrogel classification.

There are different ways of classifying a hydrogel; they can be through their (i) source, (ii) configuration of chains, (iii) polymeric composition, (iv) cross-linking type, and (v) network electrical charge [33] (Figure 2).

#### 2.1.1. Source

Cross-linked polymers are referred to as hydrogels, whether derived from natural polymers, synthetic grafts, or both. Natural hydrogels (polymers of natural origin) have the characteristic of nontoxicity and are commercialized at a meager cost. They are generally extracted from natural sources such as seaweed, brown algae, bacterial culture, diverse polysaccharides (e.g., agarose, hyaluronic acid, and alginate), and proteins (e.g., collagen, gelatin, and fibrin) and decellularized extracellular matrix. Most are biocompatible, so they are widely used in tissue engineering. However, they lack the desired mechanical properties and can trigger immune responses when introduced into the human body [34]. In contrast, synthetic polymer hydrogels can be designed with established mechanical properties. However, they do not have any inherent bioactive properties; synthetic hydrogels encompass synthetic polymers that have more flexibility to couple the mechanical properties of the hydrogel [35]. The most widely used synthetic polymers are polycaprolactone, poly(vinyl pyrrolidone) (PVP), poly(lactic acid) (PLA), poly(ethylene glycol) (PEG), and poly(vinyl alcohol) (PVA) [36].

#### 2.1.2. Configuration of Chains

Synthetic hydrogels are generally amorphous in nature without any order at the molecular level, in contrast to biological gels containing ordered aggregates at the structural level [37]. Based on the structure composition, hydrogels have three types: amorphous, semicrystalline, and crystalline [38]. In crystalline hydrogels, the molecules are arranged in a three-dimensional order and have equal intermolecular forces. Crystalline hydrogels attribute exceptional mechanical strength and flexibility to various adverse conditions, including charged biopolymers, extreme acid/base environments, and unpredictable thermal shift conditions [39]. An irregular arrangement of molecules is observed in amorphous hydrogels, and the intermolecular forces are not equal and variable in the distance between the molecules that form the hydrogel. The hydrophilic polymer has not been cross-linked in the amorphous hydrogel and remains in a gel-like state. Various amorphous hydrogels contain additives such as collagen, calcium alginate, or CMC to be more absorbent [40]. Finally, semicrystalline hydrogels are a complex combination of amorphous and crystalline phases [41].

The hydrophilic nature of hydrogels is due to the presence of functional groups along the configuration of chains. When a hydrogel undergoes drying, the water evaporates, causing the structure to collapse. The gel passes from an expanded state, of maximum swelling, to a collapsed state with the consequent expulsion of the solvent. This process is reversible, in the dry state, called xerogel, the gel is a solid material, but in the presence of water, it expands until it reaches equilibrium swelling. This latter state results from a balance between the osmotic forces that cause water to penetrate between the hydrophilic polymer chains and the cohesive forces between these chains that resist expansion [42].

#### 2.1.3. Polymeric Composition

Based on the preparation method of a hydrogel, these can be divided into (a) homopolymeric hydrogels, (b) copolymeric hydrogels, and (c) interpenetrating hydrogels (IPN) [33]. Homopolymeric hydrogels are those hydrogels of polymeric networks created based on a particular monomer type, an essential structural element that encompasses any polymeric network. These hydrogels may comprise cross-linked skeletal structures depending on the character of the monomer and polymerization methods [43]. On the other hand, copolymeric hydrogels usually contain two or more distinct monomeric species with at least one hydrophilic monomeric constituent, assembled in a random, blocky, or alternating structural configuration along the red polymer chains. In addition to the cross-linking agent, these hydrogels are made up of two or more monomers that can be polymerized to form chains of random composition or structural blocks. This type of hydrogel can be synthesized when it is desired to incorporate or improve a specific property of one of the monomers in the resulting material, such as hydrophilic character and/or sensitivity to pH [44]. Finally, IPN hydrogels are defined as a polymer comprising two or more partially interlocked networks on a molecular scale with no covalent bonds between them and that cannot be separated except by breaking chemical bonds [45].

#### 2.1.4. Cross-Linking Type

Depending on the cross-linking, they are distinguished physically and chemically as cross-linked hydrogels. Physical hydrogels are reversible and are formed by noncovalent bonds; chemical hydrogels are more stable and irreversible and are formed by covalent bonds between polymer chains. Polymer networks must obey certain conditions to form hydrogels: (a) interchain solid interactions to form stable coupling in the molecular network; and (b) the polymer network must encourage access and residence of water within the hydrogel [46]. Hydrogels that meet these demands can be prepared by noncovalent approaches such as electrostatic, hydrogen bonding, and hydrophobic forces between polymer chains. The hydrogels formed by these interactions are uniquely physical gels with high water sensitivity and thermo-reversibility [22].

Chemical or permanent hydrogels are covalently cross-linked; chemical cross-linking usually produces more stable hydrogels with better mechanical properties than physical hydrogels [25, 47]. Depending on the cross-linking type, chemical hydrogels can be obtained by radical polymerization, the reaction of functional groups, reagents, or enzymes [48, 49]. Chemical cross-linked hydrogel networks are easier to control than physical hydrogels because their synthesis and applications are not pH-dependent [50]. Furthermore, chemical cross-linking can transform the hydrogels' physical properties. Typically, swelling behavior, biodegradability, and mechanical strength have been modulated by covalent cross-linking, which can be carried out by numerous approaches [51]. For example, chemical cross-linking of polysaccharides (e.g. starch, alginate, chitin, chitosan, cellulose, oligopeptides, and hyaluronic acid) and proteins (e.g., albumin and gelatin) is typically comprised of glycosidic and amino acid repeating units, respectively [25]. Therefore, there is a wide variety of well-defined hydrogels [52].

Physically cross-linked hydrogels present cross-links between polymer chains that are formed through attractive forces or interactions, such as hydrogen bonding or hydrophobic interactions. The formation of physical gels by the clustering of molecules causes the formation of free chain loops and thus inhomogeneities, which means short-lived lattice imperfections. This type of hydrogel has an advantage over chemical cross-linking methods in that they do not require chemical cross-linking agents, initiators, or photo-irradiation that could affect the integrity of cells or proteins. However, the main drawback of physically cross-linked hydrogels is their relative instability and possible rapid and unpredictable disintegration [53].

The cross-linked structure of the hydrogel can be achieved depending on the synthesis process or the raw materials used. Physical hydrogels present reversible networks due to the weakness of their interaction; meanwhile, chemical hydrogels present permanent networks, although with a minimum degree of swelling. Currently, the synthesis of both types of hydrogels has been widely reported because each of them shows favorable characteristics [52].

#### 2.1.5. Network Electrical Charge

Hydrogels are also called “polyelectrolyte gels” when they have acidic or basic pendant groups such as carboxylic acid, sulfonic acid, primary amine, and quaternary ammonium salt [54]. Based on the network electrical charge, we have the following: (i) neutral hydrogels, also known as nonionic hydrogels. There is no charge on your spine or side meetings and due to the collaboration of water polymers, the nonionic hydrogel gradually swells in a liquid medium; (ii) anionic hydrogels have a negative charge. Hydrogel demonstrates more swelling in neutral to basic solutions due to dissociation at higher pH; and (iii) cationic hydrogels are positively charged cationic hydrogels. The predominant swelling is shown in acid media since its separation of chains is favored at low pH values [55, 56].

### 2.2. Hydrogel Swelling and pH-Sensitive Behavior and pH

Swelling is measured in terms of swelling ratio, the swelling weight ratio of swollen gel to dry gel. The swelling response in different matrix environments, such as water, pH, and ionic strength, is the characteristic of hydrogels to be used in various fields. The hydrogel responds to biological and environmental media, such as pH, ionic media, solvent, electric field, light exposure, and temperature [57]. The swelling kinetics and equilibrium are affected by different factors, such as the cross-linking ratio, the chemical nature of the polymers, the ionic media, and the synthesis state. The swelling of hydrogels is also affected by temperature and pH. pH-sensitive hydrogels swell due to the ionization of hydrophilic groups with variations in pH levels [58].

The pH-sensitive polymeric hydrogels restrict the pendant acidic and basic groups. Polymers containing acid groups, such as carboxylic acid and sulfonic acid, are known as anionic polymers, while polymers consisting of basic groups, such as amines, are cationic polymers. These groups accept or release protons and ionize with changes in pH [59]. Polymers that include a large number of such groups are recognized as polyelectrolytes. Furthermore, three-dimensional cross-linked polyelectrolytes show changes in swelling behavior with changes in pH. Pendant ionization in polyelectrolytes tends to cause differences in the corresponding acid or base's apparent dissociation constants (Ka). The swelling property of cross-linked polyelectrolytes is mainly due to the electrostatic repulsion of the ionized groups. Variations in pH, ionic strength, and electrostatic repulsion affect its properties. pH-sensitive hydrogels have been exploited for targeted and controlled oral drug release formulations [60].

For example, some studies have investigated the development of a new drug delivery system to decrease side effects and increase the efficiency of drug delivery based on the manufacture of hydrogel beads for use as a basil seed mucilage (BSM)-based drug delivery system and sodium alginate (SA) [61]; as well, another study developed a pH-sensitive hydrogel system based on fenugreek seed mucilage, extracted from *Trigonella foenum-graecum* seeds, to improve the oral bioavailability of methotrexate (MTX) [62]. In the same line of research, research has studied contact lenses as drug delivery media and the effect of physiological tear pH on drug release behavior [63]. pH-responsive dried hydrogels have been developed to indicate the healing degree of a wound [64]. Transdermal delivery systems for skin injury are being evaluated based on the physicochemical properties of pH-sensitive hydroxyethylcellulose (HEC)/hyaluronic acid (HA) complex hydrogels [65]. Various studies show how hydrogels can release, in a controlled manner, various drugs, factors, and physiological molecules in biomedical applications.

## 3. Hydrogel as an Alternative Biomaterial for Various Biomedical Purposes

In general, 2D cell cultures predominate in scientific research due to their relatively low cost (compared to others) and easy manipulation. In addition, they are not as complicated to work with as three-dimensional cultures. However, cell cultures based on three-dimensional structures are gaining popularity since they are more natural biochemical and biomechanical microenvironments [66]. Currently, there are challenges in three-dimensional cultures, such as the cellular extracellular matrix (ECM) interface, the mechanical microenvironment, spatiotemporal distributions of oxygen, and gradients of soluble factors and metabolic molecules [67], demonstrating that we are in the presence of a new and promising alternative for the biomedical research field. Actually, cell cultures, based on hydrogels, are used as three-dimensional scaffolds that provide structural integrity [68], serve as adhesive structures, tissue barriers [69], drug deposits [70], and deliver bioactive agents that promote the tissue repair process [71]. Various studies have shown that hydrogel cell cultures represent 3D environments that allow cells to behave in a manner very similar to that observed *in vivo* conditions; thus, it would be a much more realistic model in physiological terms [72].

Hydrogels in regenerative medicine have been used as scaffolds to provide structural integrity and volume for cellular organization and morphological orientation, serve as tissue and bioadhesive barriers, act as drug reservoirs, deliver bioactive agents that foster the natural repair process, and encapsulate and deliver cells [73]. These cross-linked polymer chain structures possess high water content, easy transport of oxygen, nutrients, and waste, and realistic transport of soluble factors [74]. In addition, many hydrogels can be formed under mild, cytocompatibility conditions and are easily modified to possess ligands of cell adhesion, desired viscoelasticity, and degradability [75]. Although the water content is adjustable, as well as the gelation time and degradation due to the high water content and its physical and mechanical properties, hydrogels are very appropriate for applications in soft organs such as the brain [76].

A hydrogel has particular characteristics, highlighting its soft and elastic properties. They swell in the presence of water, considerably increasing their volume but maintaining their shape until reaching a physicochemical balance [77]. They are increasingly used as biomaterials because they are highly hydrophobic and can be loaded with different soluble molecules. Their biocompatibility is currently being studied in various human tissues [78, 79]. The unique physicochemical properties of hydrogels have aroused particular interest in their use for drug delivery applications and trophic factors [80]. The structure of the hydrogel is highly porous [81, 82]. With this, its hydrophilicity and the density of its intermolecular bonds can be easily adjusted, allowing it to be loaded with various specific molecules that are released systemically [77, 83].

Hydrogels share many fundamental physical properties with native tissues, such as high water content, the same range of elasticities, and mass transport [84]. Recent advances in hydrogel assembly and generation techniques have allowed the design of new three-dimensional (3D) scaffolds and microenvironments based on physiological microenvironments [85]. Moreover, many hydrogels are highly cytocompatible and easily adapted to possess cell adhesion ligands, stable viscoelasticity, and desired degradability [86]. The properties of hydrogels that must be considered in this postinjury environment are porosity, chemical composition, and mechanical properties. The hydrogel porosity is essential to stabilize the postinjury environment by allowing the flow of nutrients in and out of the scaffold. Porosity also affects cell infiltration [87], cell distribution, cell growth, proliferation, vascularization, and local angiogenesis [88]. Thus, hydrogels are injectable materials with *in situ* formation capacity; they are cytocompatible and support cell adhesion [89]. Today, hydrogels are employed for clinical applications such as wound healing and bone regeneration; more recently, they are used as scaffolds to provide a biomimetic 3D microenvironment for brain injuries [77, 90–92].

The mechanical properties that are sought in the design of these biomaterials are the appropriate integrity and strength because, for its application in tissue engineering, the hydrogel will have to provide functions of maintenance of loads or volumes. In addition to being able to transmit mechanical stimuli to the cells for their subsequent differentiation and development of tissues, the mechanical properties highlight viscoelasticity, which is essential in the design of hydrogels because hydrogels have shown unique abilities to mimic and interact positively with the injured tissue [93, 94]. Also, rigidity is considered a fundamental factor in cell viability, demonstrating that in cell cultures, the greater the rigidity of the hydrogel, the faster cell proliferation occurs [93], as well as the mechanosensing of the stiffness of their microenvironment, which affects cell differentiation through gene expression derived from the adhesion process of cytoskeleton proteins that are capable of tuning fates [95].

In addition, it should be considered that the mass transport properties are of great importance in the generation of hydrogels, as they must allow the appropriate transport of nutrients, metabolites, gases, and cells through the scaffold. The hydrogel design must be biocompatible, a fundamental requirement for promoting the adhesion of cells in scaffolding and the subsequent proliferation, migration, and cell differentiation [96]. Also, biodegradability is a fundamental property in the design of hydrogels for the release of drugs or particles; a hydrogel's biodegradability will define the degree of administration of the substance, as well as the degradation of the remaining hydrogel in the body [31]. In addition, hydrogels can encapsulate and deliver cells; this strategy is a technological procedure with the potential to treat a wide range of human diseases by replacing damaged or diseased cells. For this reason, cell encapsulation and delivery for cellular therapy have been proposed to treat different pathologies, including chronic skin lesions, bone damage, and brain injury [89].

Hydrogels are also used in the biomedical industry for therapeutic delivery; intraocular lenses, contact lenses, and corneal prostheses in ophthalmology; bone cement for orthopedics; wound dressings; 3D tissue scaffolds in regenerative medicine; self-healing materials; biosensors; and hemostasis bandages [26, 97], due to their excellent properties such as biocompatibility, water absorption, and adequate mechanical performance, among others [98]. Furthermore, when enhanced with nanofibers and peptides, they can be used as drug delivery systems. Recent research has been conducted on the feasibility of applying hydrogels in tissue engineering and drug delivery fields.

**Figure 3:**
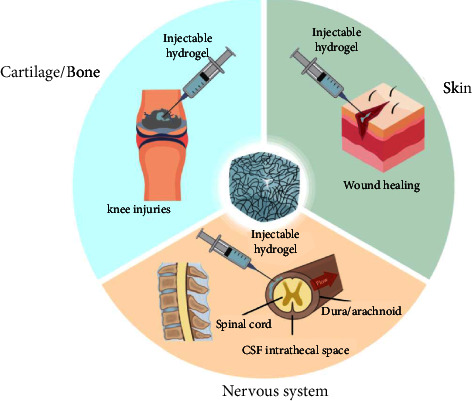
Applications of hydrogels.

In most cases, hydrogels are used as homogeneous soft materials with uniform volume properties. To better mimic body tissues' anisotropic and complex structures, hierarchical hydrogels containing multiple layers with different biochemical signals and mechanical properties play a crucial role in biomedical applications. In addition, multilayer hydrogels, formulated by combining multiple cell types, drugs, or other exogenous factors, could account for controlled release behavior or cellular interactions [99]. Hydrogels are today among the most promising materials for tissue engineering scaffolding (Figure 3). The development of hybrid hydrogels consisting of different polymers is based on numerous resources and has been applied to regenerative medicine, tissue engineering (including bone, cartilaginous, and meniscus regeneration, skin wound healing, and nervous system injuries), drug delivery, and bioelectronic interfaces [100, 101].

In summary, developing hydrogels for medical applications consists of three steps. The first step in the generation of hydrogels is the generation and characterization of the physicochemical cross-linking process; the second step is designing the response to specific stimuli (temperature, pH, ionic strength); and the third step is developing complex hybrid materials (e.g., PEG-PLA interaction) with a broad spectrum of tunable properties and trigger stimuli [82]. This last stage aimed to develop so-called “smart hydrogels” with various possible applications [102]. Hydrogel applications based on both natural and synthetic polymers offer endless possibilities to repair and regenerate a wide variety of tissues and organs [59], can respond to biological signals *in vivo* or remote triggers, and many other possible applications in biomaterials, biomedicine, and nanomedicine [82]. The ability of hydrogels to form *in situ* is advantageous for treating tissue lesions since, in their liquid phase, they can be administered through syringes (meaning a less invasive application) that allow them to fill areas with irregular lesions as opposed to solid biomaterials. This liquid-form administration can be accompanied by delivering soluble factors that directly restore tissue function to the injured area [29, 103]. Therefore, hydrogels can be particularly useful in complex environments that require advanced and invasive techniques to reach them, such as the central nervous system [104]. The main *in vivo* applications of hydrogels are in the controlled release of drugs in cartilage and bone diseases, the use of injectable hydrogels in wound healing and dressing, and the treatment of severe burns and ophthalmologic pathologies [105].

In this context, various applications in biomedicine are observed. For example, in bone regeneration, studies have shown that hydrogels provide ideal environments for bone tissue to regenerate through cell therapy. Actually, the use of injectable hydrogels in bone injury allows for filling in the defective cavity; this is a great advantage from the biomedical point of view in irregular and large lesions [106]. It has been studied *in vivo* to improve bone density in sites of bone graft donations, which do not require a lot of mechanical strength. Biomolecule-loaded hydrogels have been demonstrated to promote bone regeneration, with gels loaded with chitosan, hydroxyapatite, and osteopontin. Due to its beneficial implantation methods (through injections), hydrogels have been designed to support fracture healing, long bone defects, cartilage defects, and osteoarthritis [90, 106].

Also, a series of studies of applications of hydrogels are in wound healing, burns/scalds, and diabetic foot. Wound healing is a complex physiological process broadly divided into four phases: hemostasis, inflammation, proliferation, and degeneration [107]. Injectable gels, both natural and synthetic, are being used for wound healing and skin regeneration for implantation purposes. Among the natural-based hydrogels, alginate is one of the main biomaterials used to heal damaged skin tissue. Composites with collagen, chitosan, and gelatin have been shown to improve skin regeneration processes [108]. Synthetic polymer-based hydrogels commonly used include poly (vinyl alcohol), poly (ethylene glycol), polycaprolactone, and polylactic acid. These are used to fabricate scaffolds capable of cell attachment and growth in cutaneous regeneration [109]. Currently, hydrogels are very popular as a therapeutic strategy in the delivery of antibiotics, antiseptics, antioxidants, and anti-inflammatories in the treatment of various skin wounds. In addition, hydrogels can be modified and studied to target wounds' specific pathophysiology since, depending on the type of wound, the origin, and the patient's condition and characteristics, the approach to wound dressing, healing, and repair must be different [91, 110]. Currently, several types of new hydrogel dressings are being continuously optimized, and their functions are progressively becoming ideal. However, there is still a gap in the ideal hydrogel depot, especially in treating chronic wounds.

According to various authors, the most challenging issue today is the generation of functional biomaterials to recover from nervous system injuries. The most crucial step in material bioengineering is brain repair by developing scaffolds that contain the mechanical, topological, and biochemical properties necessary to promote axonal regrowth in an injured brain [104]. The main advantage offered by hydrogels is that they have biomechanical and molecular properties that are compatible with the adjacent areas of injured tissue [18]. It is well known that the brain has a minimal capacity for tissue repair in the face of any damage (e.g., trauma, neurodegenerative diseases), thus resulting in a permanent loss of nervous tissue [111]; that is why one of the biggest challenges in the scientific field of research in biomedicine is to find solutions to brain injuries [112]. One strategy is the search for artificial supports, such as hydrogels, where cells can proliferate, migrate, and differentiate as if they were part of the original tissue. In addition, various biomolecules, growth factors, and neurotrophins could be added to the biomaterial to help the recovery of injured brain tissue [34].

In the context of brain injuries, different hydrogel biomaterials based on materials such as alginate, gelatin, chitosan, methylcellulose, collagen, hyaluronic acid, and PLGA [113] have been used as microcarriers for the release of growth, angiogenic, and neurotrophic factors [114]. Currently, several well-documented studies show that stem cell-based therapy may be able to reduce the size of cortical infarction and increase blood vessel density [115]. However, cell transplant strategies often have low cell survival as their main drawback because the surrounding environment at the injury site has a series of signals capable of inhibiting the regenerative process [116]. Therefore, a possible way to address this problem is to encapsulate the cells to be transplanted into suitable biomaterials capable of protecting the cells in the implantation area, which can support cells [117].

Due to its many applications in the healthcare field (as shown in Table 1), this is the leading industry responsible for the increased market value of these biomaterials worldwide. Hydrogels have been a revolutionary option developed for contact lenses for the last 60 years, which is why they have become the biggest client for hydrogels, according to BCC Research. Moreover, it is expected that the wound-care field will become a rapid-growing representative for hydrogel applications in the next ten years, along with the cosmetic, skincare, and tissue engineering industries [118–120]. In 2016, the global hydrogel market reached a total of $15.6 and was predicted to increase by a 6.3% compound annual growth rate (CAGR) (meaning a final value of $22.3 billion), as described by BCC Research [120]. This prediction was proved correct by Precedence Research [118]. When the 2021 market value totaled $22.45 billion by the end of that year and the beginning of 2022, the expected market value is set to maintain a 6% increase from 2022 to 2030. Exceptionally well-known companies dedicated to pharmaceutics, medicine, and research are now some of the main producers and sellers of commercial hydrogels, such as Johnson & Johnson Services, Medtronic, Medline Industry, Advanced Biohealing, Inc., Organogenesis, Foryou Medical, Hisamitsu Pharmaceutical Co., Coloplast, and 3M Company, among many others [91, 121].

## 4. Discussion

Hydrogels are used in many fields of the biomedical industry, such as therapeutic administration [145, 146], bone cement in orthopedics [147], wound dressings in nursing [148], and tissue scaffolds in regenerative medicine [149], due to their excellent properties such as biocompatibility, water absorption, and adequate mechanical performance, among others [98]. In the pharmaceutical branch, hydrogels are being studied as drug delivery systems, consisting of hydrogels designed to load drugs inside for therapeutic administration. The design of hydrogels would specify their degradation characteristics depending on the type of drug release that is necessary; being hydrogels, the main advantage of this application is that the administration of the hydrogel can be made by local injection, intravenous infusion, or surgical implantation, and once the drug is implanted and released, the hydrogel is designed to degrade in the body and prevent its extraction through additional clinical procedures [26, 97]. In most cases, hydrogels are used in tissue engineering to mimic the anisotropic and complex structure of body tissues through different biochemical signals and various mechanical properties that play a crucial role in various biomedical applications [149]. For example, there are studies in which hydrogels can act as implantable devices and be loaded with bioactive agents, allowing the controlled release of hormones, such as insulin [99].

Improving mechanical properties is one of the most desirable achievements in hydrogel engineering, and many researchers are currently working in this complex scientific area. However, most hydrogels possess only shallow mechanical properties, especially in a swollen state [150]. More recent studies have demonstrated new procedures to improve the mechanical properties of hydrogels by incorporating nanomaterials, such as graphene and its derivatives (e.g., carbon nanotubes and graphene oxide) [26, 98]. Several techniques have been developed to manufacture hydrogels that include microscale ordered particles (1–500 *μ*m), called microgels or microparticle-based hydrogels (HMP) [96]. HMPs can be made from natural or synthetic polymers and manufactured in a variety of shapes and sizes using techniques that are often compatible with the encapsulation of biologics (e.g., cells and drugs) [151], and they can be administered by intra-articular injection or direct injection into tissues [152]. For many years, effective and reliable oral insulin delivery strategies have been sought to treat diabetes. Currently, hydrogels are being explored in oral insulin administration as it protects and prevents the degradation of the peptide during its passage through the stomach and allow its release into the bloodstream [153].

Hydrogels are used in many fields of the biomedical industry, such as therapeutic delivery, intraocular lenses, contact lenses, corneal prostheses in ophthalmology, bone cement for orthopedics, wound dressings, and 3D tissue scaffolds in regenerative medicine, due to their excellent properties such as biocompatibility, water absorption, and adequate mechanical performance, among others [98]. In most cases, hydrogels are used as homogeneous soft materials with uniform bulk properties. However, hierarchical hydrogels containing multiple layers with different biochemical signals and various mechanical properties play a crucial role in biomedical applications to better mimic the anisotropic and complex structure of body tissues.

Besides the already-known characteristics of hydrogels and their current uses, plenty of upcoming investigations promise to reach the full potential of these materials. For example, we can mention the use of chitosan for the administration of growth factors; a hybrid material made from chitosan-polyvinyl alcohol-epichlorohydrin with high regenerative prospects for elastic cartilage [154]. Similarly, these novel hydrogels can be used as fibers for actuators, artificial adhesives, transplantable tissue organs, and soft tissue recovery treatments, which is crucial to counteract the limitations of standard treatment to more effectively promote reparative dentinogenesis [155]. In summary, hydrogels can provide different biological enhancement insights in designing rigid tissue scaffolds for orthopedic insertion injuries. Thus, injectable hydrogels are promising scaffolds for cartilage and bone tissue engineering, owing to their minimal invasive properties and ability to match irregular defects.

Various methods for treating chronic wounds include debridement, hyperbaric oxygen therapy, ultrasound, electromagnetic therapies, negative pressure wound therapy, skin grafts, and hydrogel dressings. The intrinsic characteristics of hydrogels allow them to benefit from cutaneous healing essentially by supporting a moist environment. Hydrogel-based systems offer tremendous potential in skin regeneration for several reasons: they have long been recognized as the standard treatment for necrotic and sloughy wounds [156]; they offer sustained and/or stimuli-responsive delivery of antiseptics or antibiotics [157]; they can modulate the local inflammatory environment or incorporate antioxidants or anti-inflammatories [158]; and incorporating bioactive agents and different types of cells have been proposed as the future of advanced therapies [110]. Hydrogel dressings are more advantageous for chronic wound treatment because they can be conveniently designed to meet the specific needs of the treatment of chronic wounds. Hydrogel dressings can promote wound healing by improving autolytic debridement, tissue hydration, and wound oxygenation. This helps to recover lost skin capacity after tissue damage. The main hydrogels used in skin regeneration are derived from chitosan, agar, gelatin, and methylcellulose [108]. Conversely, ethnomedicinal plants can treat wounds as they have no side effects, while in the case of chemical drugs, side effects are increasing. Based on this knowledge, the increase in applications of hydrogels of vegetable origin has now begun, for example, *Moringa oleifera, Aloe vera,* and others [159–161].

Another notable advance in biomedicine is photo-cross-linkable hydrogels since their properties can be manipulated in space-time through exposure to light to achieve desirable parameters translated into multiple biomedical applications (critical in tissue engineering), regenerative medicine, and drug delivery [162]. Hydrogels have become the allies of regenerative medicine recently. The advantages of the different rigidities of the different hydrogels and their impact on various treatments and applications, significantly influencing the treatment of stem cells (BMSC), are being studied [163]. Additionally, researchers propose simulating muscle, skin, and dermis tissue with the help of 3D hydrogels to maximize the rigidity qualities of the hydrogel [164]. It can be concluded that although the applications of hydrogels have been attenuating for a few years, there is still an extensive breakdown of possibilities for using hydrogels for multiple applications in areas such as regenerative medicine, tissue engineering, and many others.

An interesting approach to hydrogels has been their use to repair CNS injuries, mainly due to the properties mentioned before and their ability to be implanted through minimally invasive procedures for drug delivery purposes. To try to imitate the unique composition of tissues, several techniques have been developed to capture the microenvironment *in vivo* of tissues by employing the decellularization technique [165]. In addition, decellularized tissues and organs have become increasingly frequent in studies in cell culture and animal models [166]. The technology of hydrogels based on the decellularized extracellular matrix is an attractive approach for the treatment of CNS lesions because it has multiple positive effects, such as biocompatibility, immunomodulation, and tissue integration capacity, which provides a mimetic, minimally invasive platform that can be adjusted both mechanically and biochemically to deliver to the environment where various bioactive, angiogenic, and growth factors are implanted [167], as well as cells [168].

Recent studies integrate stem cells into neural cells by embedding them into native tissue hydrogels, such as ECM hydrogels. ECM-based hydrogels implanted in the brain cavity that have suffered tissue loss have demonstrated the ability to attract endogenous cells. Furthermore, these 3D gels can be formulated at different concentrations of proteins, allowing the control of their rheological and inductive properties [169]. All this will enable us to infer that, in the case of nervous system regeneration, the components that make up the brain microenvironment may be necessary for neuronal maturation and differentiation [170, 171].

Several techniques have been developed to mimic the unique composition of tissues. That closely captures the microenvironment *in vivo* using decellularized tissues or cell-derived ECM proteins. Using decellularized tissues and organs has become increasingly prevalent in preclinical animal studies and human clinical applications [172]. ECM hydrogel technology is an attractive approach for treating CNS disease and trauma. Two fundamental steps are required for ECM hydrogel formation: (i) ECM material solubilization into monomeric protein components and (ii) neutralization controlled by temperature and/or pH to provoke instinctive reformation of the intramolecular bonds of the monomeric components in a hydrogel [173]. The water content, gelation time, and the degradation rate are all adjustable. Due to their high water content and physical and mechanical properties, hydrogels are highly appropriate for soft organs such as the brain [3].

In addition to the favorable biocompatibility, immunomodulation, and tissue remodeling effects of naturally derived ECM scaffolds, ECM's hydrogel technology provides a flexible platform that can be adjusted mechanically and biochemically to multiple implantation environments [127, 174]. Factors, including cells, growth factors, and other bioactive factors can be delivered through minimally invasive injections. Initial studies have shown upbeat delivery and remodeling of tissues in animal models of CNS injury [174, 175]. In addition, decellularized brain ECM from animal models is reported to be compatible with neural cells as induced pluripotent stem cell-derived neurons grow and mature on such scaffolds. In recent work, cortex-derived neural networks were more abundant when implanted within decellularized brain ECM scaffolds than type I collagen and Matrigel controls [176, 177].

There has been a growing interest in transplanting different stem cells to restore neurological functions and improve behavioral recovery after an ischemic attack. Several well-documented studies show that transplanted stem cells can enhance ischemic stroke by reducing the cortical infarct size and increasing the blood vessels' density. However, cell transplantation strategies often suffer from poor cell survival due to the toxicity of the surrounding environment at the injury site. One possible way to address this problem is by encapsulating the cells to be transplanted in suitable microenvironments that protect the cells during and after implantation and support cell growth and survival. In addition, the engineering and design of the biomaterials to be used can influence transplanted or endogenous stem cells' subsequent fate by directing their differentiation; this is when hydrogels are studied and designed as scaffolds for stem cell implantation and their subsequent differentiation [175].

Thus, hydrogel studies' future is going towards regenerative medicine through stem cells in various organ systems. Even though it is uncertain that these applications will be suitable for clinical trials, the available literature suggests that the road researchers are taking to study and develop hydrogels is the right one. So far, the results make them a promising biomaterial for future research concerning bioengineering and regenerative medicine.

## 5. Conclusions

Hydrogels are an essential tool for regenerative medicine and biomedical engineering; their applications have been and are still being tested in most organ systems due to the promising results already obtained. These biomaterials are so widely studied that their properties include water absorption, biocompatibility, and the ability to mimic native tissue for cell proliferation and differentiation. Some promising biomaterials are light-sensitive hydrogels that have been researched for possible application in drug delivery, microlenses, and biosensors due to the remote and noninvasive nature of their activation process. This hydrogel type has promising roles in bioengineered applications to release molecular and cellular species. These materials offer real advantages to the organizational engineering community and will likely attract increasing attention in the coming years. In the future, researchers hope to explore the functions of various hydrogel derivatives in biomaterial formulations, which will be an excellent opportunity to develop new materials with new properties.

Furthermore, different hydrogel materials can be specially modified and functionalized to achieve better performance. Hydrogels' exploitation is constantly increasing after evidence of their even broader therapeutic potential due to their ability to induce tissue regeneration. The innovation in advanced tissue repair is further directed to the development of so-called smart hydrogels, which combine hydrogels with components that enhance the primary purpose of providing a beneficial environment for tissue regeneration.

Hydrogels have shown the advantage of allowing researchers to modify their physical and chemical properties to meet each study's needs. Thus, hydrogels are great candidates for vastly different applications besides the hundreds of known commercial applications that are responsible for their exponential growth in different markets. In addition, many newly developed hydrogels perform well *in vitro* tests but not so well *in vivo*. From *in vitro* experiments to *in vivo*, we need a more systematic strategy to develop strategies for advanced biomaterials.

## Figures and Tables

**Table 1:** A summary of some of the main applications of hydrogels in biomedicine and regenerative medicine.

Hydrogel material	Applications	Ref.
*Synthetic*		

Polyvinyl alcohol (PVA)	(i) PVA hydrogel seeded with adipose-derived stem cells for wound dressing, promoting wound healing through stem cell delivery *in vitro* and *in vivo*.	[122]

Poly (ethylene glycol) (PEG)	(i) Tuning of alginate and PEG hydrogels allows fibroblast proliferation and increases osteogenic differentiation of MSCs. (ii) Photoclickable PEG hydrogels as a 3D cell culture scaffold for adult mouse cardiomyocytes, exhibiting biomimetic physiological and pathological microenvironments	[123, 124]

*Naturals*		

Hyaluronic acid (HA)	(i) Peptide-modified HA hydrogels to culture NSCs. Resulting in increased differentiation towards oligodendrocytes and neurons over 2D cultures on laminin-coated glass. (ii) Localized delivery of therapeutic cargo to CNS damage areas. (iii) Local delivery of BDNF on brain injury sites provoked by stroke.	[125–127]

Chitosan	(i) Transplantation of bone marrow mesenchymal stem cells and collagen-chitosan scaffolds to traumatic brain injury rats. (ii) Capacity to stand sufficient physiological activity of primary cultured neuronal cells. (iii) Skin infection control. Improved antimicrobial effect against *S. aureus* and *S. epidermidis*	[128, 129]

Collagen	(i) Three-dimensional collagen/silk fibroin scaffold (3D-CF) with cavities that simulate the anatomy of normal spinal cord. (ii) Wound dressing for refractory skin wounds accelerates the healing of deep second-degree burn wounds and the generation of new skin appendages. (iii) Nervous regeneration. *In vitro* study for peripheral nerve damage treatment. (iv) Bone tissue engineering. By developing a bone graft structure that can heal bone defects, having *in vitro* results of cellular proliferation and *in vivo* results of potential bone healing hydrogel. (v) Tissue regeneration in skin defects. Fabrication of bio-nano hydrogel systems demonstrating rapid tissue regeneration at the wound site in *in vivo* tests.	[130–133]

Alginate	(i) Neuroprotector for ischemic brain cells. (ii) Development of electroactive nanocomposite hydrogel. *In vitro* tests proved PC12 cells proliferate and spread evidently. (iii) Bone regeneration. Sustained delivery for bone regeneration in osteoporosis by *in vitro* and *in vivo* analysis	[134–136]

Fibrin	(i) Cavity repair resulting from a stroke. (ii) Neural progenitor cell delivery in spinal cord injury. Cell/neural tissue-compatible biomaterial for improving NPC *in vivo* survival.	[137, 138]

Gelatin	(i) Drug delivery. Improves wound healing and skin flap survival by the sustained release of basic fibroblast growth factor. (ii) Skin protection. Excellent UV protection properties and broad absorption of UV across UVA and UVB regions. (iii) Nerve regeneration scaffold. Ideal microstructure to prevent fibrous tissue ingrowth into the injury site.	[139–141]

Decellularized matrices	(i) Neural stem/progenitor cell microenvironment reconstruction, axonal regeneration, and spinal cord injury. (ii) Nervous regeneration. A hydrogel derived from porcine decellularized nerve tissue to repair peripheral nerve defects. (iii) Wound healing. The hydrogel yields significantly advanced wound closure in 24 h via the human dermal fibroblast scratch model.	[142–144]
